# Critical trigger thresholds for hemostatic management: a narrative review of viscoelastic hemostatic assay applications

**DOI:** 10.3389/fmed.2025.1658845

**Published:** 2025-10-15

**Authors:** Zhi-Li Xu, Han Cao, Da-Wei Sun, Jie Xiao, Yuan-Yuan Yao, Ge Luo, Ting-Ting Wang, Qi Gao, Jing-Cheng Zou, Xin-Chen Tao, Min Yan

**Affiliations:** ^1^School of Anesthesiology, Shandong Second Medical University, Weifang, Shandong, China; ^2^Department of Anesthesiology, The Second Affiliated Hospital, Zhejiang University School of Medicine, Hangzhou, Zhejiang, China

**Keywords:** viscoelastic hemostatic assay, thromboelastography, rotational thromboelastometry, major bleeding, coagulopathy, transfusion

## Abstract

Viscoelastic haemostasis assays (VHA) are increasingly used in clinical practice. These bedside whole-blood tests, commonly implemented through thromboelastography (TEG) and rotational thromboelastometry (ROTEM), are prized for their speed, accuracy, and accessibility, resulting in their increased usage in managing complex cases such as severe trauma, cardiac surgery, postpartum hemorrhage, and liver disease. Despite their widespread use, clear guidelines for regulating coagulation function in surgical patients through VHA remain undefined. This review searched the majority of the literature on VHA in the past decade and discussed the triggers and algorithms for hemostatic management guided by VHA in surgeries with a high risk of major bleeding. It also reviews the potential benefits of VHA over traditional coagulation tests (like prothrombin time and partial thromboplastin time) and clinical judgments, focusing on aspects such as reducing bleeding volume, decreasing use of allogeneic blood products, improving patient outcomes and mortality, and enhancing cost-effectiveness.

## Background

Hemorrhage is one of the main causes of death and various complications in severe trauma and major surgeries (including cardiac surgery, organ transplantation, and obstetric delivery). 40% of trauma patient deaths and 10% of cardiac surgery patient deaths are due to hemorrhage, while maternal hemorrhage accounts for 30% of maternal mortality (1–3). Major bleeding can cause multiple pathophysiological responses such as tissue damage, endothelial irritation, immune system activation, platelet dysfunction, and coagulation disturbances, leading to bleeding-related coagulopathy and resulting in early and late hypercoagulability (4, 5). Ultimately, death occurs due to excessive blood loss, multi-organ dysfunction, or shock (6). Therefore, early identification and treatment of coagulopathy in patients with major bleeding or at risk is crucial for improving patient prognosis.

Conventional coagulation tests (CCT), including prothrombin time (PT), activated partial thromboplastin time and so on were primarily developed for the assessment of anticoagulant therapy and hemophilia (7). However, these tests cannot provide continuous, comprehensive, and accurate dynamic analyses of clot formation, stability, and the interactions among various blood components. Furthermore, their results can take 60 min or even longer to obtain, significantly affecting the diagnosis of coagulopathy in emergencies and may result in missing the optimal time for treatment (8–10). Viscoelastic hemostatic assays (VHA), such as thromboelastography (TEG) and rotational thromboelastometry (ROTEM), are bedside whole-blood testing devices that can dynamically display the indicators, including clot initiation time, fibrinogen levels, platelet function (9, 11), and fibrinolytic activity. They can use just 0.3–0.4 ml blood to provide coagulation-related information for clinical transfusion guidance in as little as 10 min (10, 12, 13). Studies have shown that parameters like maximum clot firmness and clotting time in ROTEM or TEG can predict coagulopathy and major bleeding (14, 15) and that ROTEM is sensitive and specific for intraoperative platelet and fibrinogen levels (16) (Table 1).

**Table 1:** Parameters of TEG and ROTEM and their normal reference ranges.

**Parameter (unit)**	**Explanation**	**Clinical significance**	**Normal reference range**
**TEG** ^*^			
R time (s)	Time from test start to an amplitude of 2 mm	Prolonged time suggests coagulation factor deficiency, may require FFP or PCC	5–10 min
K time (s)	Time between 2 and 20 mm clot amplitude	Prolonged time suggests fibrinogen deficiency, may require fibrinogen supplementation	1–3 min
α angle (°)	Slope between R and K	Decreased angle suggests fibrinogen or platelet function deficiency	53–72 °
MA (mm)	Maximum clot strength	Decreased MA indicates platelet or fibrinogen deficiency, may require platelets or fibrinogen	50–70 mm
Ly30 (%)	Percentage of clot lysis at 30 min	Increased Ly30 suggests increased fibrinolysis, may require antifibrinolytic agents like TXA	0%−8%
**ROTEM**			
CT time (s)	Time from test start to an amplitude of 2 mm	Prolonged CT suggests coagulation factor deficiency, may require FFP or PCC	38–79 s (INTEM)
CFT time (s)	Time between 2 and 20 mm clot amplitude	Prolonged CFT suggests fibrinogen deficiency, may require fibrinogen supplementation	34–159 s (INTEM)
α angle (°)	Tangential angle at 2 mm amplitude	Decreased angle suggests fibrinogen or platelet function deficiency	70–83 ° (INTEM)
MCF (mm)	Maximum clot firmness	Decreased MCF indicates platelet or fibrinogen deficiency, may require platelets or fibrinogen	50–72 mm (INTEM)
LI30 (%)	Percentage of clot lysis at 30 min	Increased LI30 suggests increased fibrinolysis, may require antifibrinolytic agents	94%−100% (INTEM)
ML (%)	Maximum lysis detected during the run time, as % of MCF	Maximal clot stability and fibrinolysis	0%−15% (INTEM)

^*^All of our TEG are designed for kaolin. R time, reaction time; K time, kinetics time; α angle, alpha angle; MA, maximum amplitude; Ly30, percentage of fibrinolysis at 30 min; CT time, clotting time; CFT time, clot formation time; α angle, alpha angle; MCF, maximum clot firmness; LI30, lysis index at 30 min; ML, maximum lysis.

A meta-analysis by Fahrendorff et al. (17) indicated that VHA-guided transfusion in various types of major bleeding surgeries can reduce total blood loss (Standardized Mean Difference (SMD) = 1.40, 95% CI: 2.57–0.23; *p* = 0.02), the transfusion of allogeneic red blood cells (RBCs; SMD = 0.64, 95% CI: 1.12–0.15; *p* = 0.01), and fresh frozen plasma (FFP; SMD = 1.98, 95% CI: 3.41–0.54; *p* = 0.01), but there was no significant statistical difference in all-cause mortality and platelet transfusion. Dias et al. (18) also noted that using VHA can reduce bleeding, decrease the use of plasma and platelets, shorten Intensive Care Unit stay, and increase ventilator-free days, but its impact on mortality remains controversial, which may be due to differences in types of surgery. Baksaas-Aasen et al. (6) demonstrated that in trauma patients receiving the massive hemorrhage protocols (MHPs)—which include empirical administration of tranexamic acid (TXA), transfusion of RBCs, plasma, and platelets (PLT) in a 1:1:1 ratio, and restricted crystalloid supplementation—there were no significant statistical differences in the allogeneic blood product transfusion and mortality when guided by VHA or CCT. Because the optimized balanced hemostatic treatment before grouping resulted in a lower-than-expected overall rate of coagulopathy in patients. Meanwhile, Santos et al. (19) believed that using VHA significantly reduces mortality compared to standard laboratory tests and/or clinical decisions (7.3 vs. 12.1%; RR = 0.64, *p* = 0.03). The aforementioned discrepancies may be primarily attributed to the fact that Baksaas-Aasen et al. employed a randomized controlled study design, whereas Santos et al. conducted a meta-analysis, with additional variations existing in surgical procedures and patient characteristics.

These reseach primarily focused on VHA-guided transfusion algorithms; however, there is less emphasis on specific transfusion triggers. Most studies currently rely on institution-specific VHA protocols for guiding transfusion, and there is a lack of high-quality evidence calculating these specific thresholds. In this review, we have compiled and listed the various VHA algorithms reported in the literature, providing a reference for clinicians to understand the current practices and guide future standardization efforts.

Many regional and international guidelines recommend VHA use in surgeries with a high risk of major bleeding, such as trauma (20, 21), cardiac surgery, obstetrics (22), and liver disease (23). However, differences in VHA-guided transfusion algorithms across surgical contexts persist, largely due to the absence of standardized trigger thresholds. Agarwal et al. (24) highlighted that VHA use during and after cardiac surgery can reduce perioperative transfusions of allogeneic blood products, but the lack of standardized thresholds limits consistency across studies. Therefore, establishing standardized VHA algorithm triggers for different surgeries is essential to optimize transfusion management.

## VHA in trauma

Trauma-induced coagulopathy (TIC) caused by trauma hemorrhage is one of the leading causes of death in patients affected by trauma and is independently associated with increased mortality (25). In severe traumatic patients, 20 to 30% of patients develop TIC within minutes after injury, leading to early mortality. It is now widely accepted in research that early identification and intervention of coagulopathy in severe trauma patients can reduce blood loss, decrease the need for allogeneic blood products, and improve patient prognosis (26). However, traditional post-trauma transfusion decisions rely primarily on clinical experience, lacking accuracy and generalizability, which leads to increased transfusion-related complications, blood product consumption, and mortality rates. Therefore, the introduction of VHA technology may provide valuable guidance for early trauma transfusion management and potentially help reduce coagulopathy-induced mortality (27). Although most studies of patients with traumatic hemorrhage have identified their respective transfusion trigger thresholds, there is still a lack of globally harmonized, high certainty of evidence of transfusion triggers' values for VHAs that can be widely used in clinical practice.

The trigger thresholds for VHA-guided blood management in trauma patients are presented in Table 2. ROTEM FIBTEM A5 ≤ 7–10 mm and TEG MA < 20 mm or citrated functional fibrinogen (CFF) MA < 14 mm are primarily used to guide the transfusion of fibrinogen, fibrinogen concentrate (FC), or cryoprecipitate (5, 10, 28). Additionally, FFP and Prothrombin complex concentrate (PCC) may be transfused when EXTEM CT > 80 s or CT > 90 s, and FIBTEM A5 ≥ 6–10 mm or rTEG ACT ≥ 120 s and MA > 65 mm (10, 29). Moreover, platelet transfusion in ROTEM is generally determined by EXTEM and FIBTEM indirectly, such as when EXTEM A5 < 35 mm and FIBTEM A5 > 9 or 10 mm (5, 30). Alternatively, it can be solely determined by FIBTEM A10/5 > 7–9 mm. In TEG, it is determined by the MA values. Furthermore, in ROTEM, TXA administration is guided by EXTEM lysis index at 30 min (LI30) < 85%, maximum lysis (ML) >15%, or FIBTEM CT > 300 s combined with EXTEM A5 < 35 mm or rTEG fibrinolysis rate (LY30) >10% (10, 29).

**Table 2:** Trigger thresholds and transfusion in trauma surgery using VHA algorithms.

**Author (year)**	**Sample size**	**Research type**	**Reduction of blood product**	**VHA**	**FC/fibrinogen/ cryoprecipitate**		**FFP/PLASMA/PCC**		**PLT**		**TXA**	
					**Trigger value**	**Dosage**	**Trigger value**	**Dosage**	**Trigger value**	**Dosage**	**Trigger value**	**Dosage**
David et al. (2023) (5)	*n* = 624	Retrospective	Yes	ROTEM	FIBTEM A5: 7–8 mm	Fibrinogen 25 mg/kg	EXTEM CT > 106 s	107–135 s → FFP10 ml/kg OR PCC10 μl/kg 136–200 s → FFP20 ml/kg OR PCC20 μl/kg > 200 s → FFP20 ml/kg AND PCC20 Ul/k	FIBTEM A5 > 9 mm	PLT	EXTEM ML > 15%	TXA 1 g
					FIBTEM A5 < 7 mm	Fibrinogen 5–6 mm= 25 mg/kg < 5 mm = 50 mg/kg	EXTEM CT > 90 s AND FIBTEM A5 > 6 mm	FFPs/PCCs				
Barquero López et al. (2022) (29)	*n* = 135	Retrospective	Yes	ROTEM	EXTEM A10 ≤ 40 mm FIBTEM A10 ≤ 7 mm	Fibrinogen: FIBTEM goal-current FIBTEM x (Kg/140) = grams needed (mm)	EXTEM CT> 80 s	PCC: 500–1,000 IU	EXTEM A10 ≤ 40 mm FIBTEM A10 > 7 mm PLT < 50.000–75.000/μl	1 PLTs concentrate	ML > 15%	TAX 1 g
George et al. (2022) (30)	*n* = 167	Retrospective	NA	ROTEM	FIBTEM A5 ≤ 10 mm	FC: 50 mg/kg	FIBTEM A5 > 10 mm AND EXTEM CT ≥ 90 s	FFP1: 5 ml/kg	FIBTEM A5 > 10 mm AND EXTEM A5 ≤ 35 mm	PLTs: 10–15 ml/kg	FIBTEM CT > 300 s AND EXTEM A5 ≤ 35 mm	TXA: 15 mg/kg + FC: 50 mg/Kg
						Cryoprecipitate:5 ml/kg		PCC: 10 IU/Kg			ML ≥ 5%	TXA: 15 mg/kg (max 1 g)
Spagnolello et al. (2021) (31)	*n* = 57	Prospective observational	NA	ROTEM	\	\	EXTEM A10 ≥ 39 mm (A5 ≥ 28 mm) AND FIBTEM A10 < 5 mm (A5 ≤ 3 mm)	FFP: 4 unit	FIBTEM A10 ≥ 8 mm (A5 ≥ 7 mm)	PLT	\	\
Cochrane et al. (2020) (28)	*n* = 301	Prospective observational	Yes	TEG	CFF MA < 14 mm AND CRT MA < 50 mm	Three pools cryoprecipitate	CRT ACT ≥ 120 s	FFP/octoplas: 12–15	CRT MA < 60 mm AND CFF MA ≥ 14 mm	PLTs one pool	CRT LY30 ≥ 3%	TAX: 1 g OR 10 mg/kg
					CFF MA < 14 mm AND CRT MA ≥50 mm	Two pools cryoprecipitate						
Baksaas-Aasen et al. (2017) (10)	*n* = 392	RCT	NA	ROTEM	FIBTEM A5 < 10 mm	4 g equivalent of fibrinogen	EXTEM A5 > 40 mm AND EXTEM CT > 80 s	4 units of plasma	(EXTEM A5 -FIBTEM A5) < 30 mm	One pool of PLTs	EXTEM LI30 < 85 %	1 g TXA
				TEG	MA < 20 mm	4 g equivalent of fibrinogen	rTEG MA ≥ 65 mm AND rTEG AC0T > 120 s	4 units of plasma	(rTEG MA- FF TEG MA) < 45 mm	One pool of PLTs	rTEG LY30 > 10 %	1 g TXA

FC, fibrinogen concentrate; FFP, fresh frozen plasma; PCC, prothrombin complex concentrate; PLT, platelet; TXA, tranexamic acid; FIBTEM, fibrinogen thromboelastometry; EXTEM, external thromboelastometry; CT, time from test start to an amplitude of 2 mm; CFF, citrated functional fibrinogen; CRT, citrated rapid TEG; ACT, TEG activated clotting time; rTEG, rapid TEG; LI30, percentage of clot lysis at 30 min; LY30, percentage of clot lysis at 30 min; A10, the clot amplitude at 10 min after the beginning of clot formation; A5, the clot amplitude at 5 min after the beginning of clot formation; MA, maximum clot strength.

A retrospective study by David et al. (5) shows that, compared to CCT transfusion algorithms, using ROTEM-guided transfusion algorithms resulted in more patients surviving without substantial blood product transfusion within 24 h, fewer patients required extensive blood product treatment (32 cases, 15% vs. 91 cases, 42%; *p* < 0.01); however, there was no significant impact on mortality rates. Conversely, the study by Cochrane et al. (28) showed that compared to CCT, patients guided by TEG algorithms had significantly reduced mortality rates at 24 h (13% vs. 5%; *p* = 0.006) and 30 days (25% vs. 11%; *p* = 0.002), and reduced blood product wastage (1.8 ± 2.1 vs. 1.1 ± 2.0; *p* = 0.002); however, there was no difference in blood product transfusion volumes. Baksaas-Aasen et al. conducted a randomized controlled study involving 396 patients, showing no overall differences in survival without massive transfusion and 28-day mortality rates between the VHA and CCT groups. However, among a predefined subgroup of 74 patients with traumatic brain injury (TBI), the VHA group had a higher proportion of survivals without massive transfusion within 24 h compared to the CCT group (OR = 2.12, 95% CI: 0.84–5.34) (6).

Although there is still a controversy regarding the use of VHA algorithms in trauma patients for the transfusion of allogeneic blood products and prognosis. However, VHA enables quicker and more comprehensive assessment of coagulation in trauma settings, facilitating earlier clinical intervention (31). Additionally, guidelines recommend using VHA algorithms to guide fluid resuscitation in patients affected by trauma (20, 21). Furthermore, using VHA for clinical blood management enables early diagnosis of TIC and prediction of blood product transfusion and mortality post-trauma (32). The use of VHA in trauma patients not only optimizes transfusion algorithms, but also provides accurate, clinically generalizable transfusion trigger thresholds that offer a viable way to address traumatic coagulopathies.

## VHA in cardiothoracic surgery

Preoperative anticoagulant and antiplatelet drugs, cardiopulmonary bypass (CPB) surgery, anesthetic administration can all cause the patient's blood composition to become unstable during cardiac surgery (33). This can result in severe bleeding that requires reoperation and poses a serious risk to patient's life (24). There is a lack of quantitative metrics for the timing and amount of blood transfusions for cardiac surgery, where the VHA's transfusion trigger thresholds have a potential role.

The algorithm for VHA-guided blood product transfusion in cardiac surgery is shown in Table 3. The transfusion of fibrinogen, FC, or cryoprecipitate is usually guided by ROTEM FIBTEM A10 ≤ 7–10 mm or maximum clot firmness (MCF) < 6–8 mm; in TEG, it is primarily determined by an α-angle of < 45–53 °, CFF MA < 12 mm, or MA < 17 mm in conjunction with citrated rapid TEG maximum amplitude (CRT MA) >47 mm. Additionally, when ROTEM EXTEM CT ≥ 80–111 s, HEPTEM CT > 240–280 s, or TEG reaction time (R) > 10 mins. FFP, plasma, or PCC can be transfused to prevent excessive depletion of coagulation factors. Platelet transfusion is primarily guided by minutes ROTEM EXTEM A10 ≤ 30–42 mm and FIBTEM A10 > 7–10 mm; HEPTEM MCF can also guide platelet transfusion (24). In TEG, intervention for platelet reduction is guided by an MA < 30–50 mm. TXA transfusion is primarily guided by ROTEM EXTEM LI30 < 90% or LI30 > 15% and TEG LY30 > 3, 7.5, or 15% to counteract hyperfibrinolysis.

**Table 3:** Trigger thresholds and transfusion in cardiac surgery using VHA algorithms.

**Author (year)**	**Sample size**	**Research type**	**Reduction of blood product**	**VHA**	**FC/fibrinogen/cryoprecipitate**		**FFP/PLASMA/PCC**		**PLT**		**TXA**	
					**Trigger value**	**Dosage**	**Trigger value**	**Dosage**	**Trigger value**	**Dosage**	**Trigger value**	**Dosage**
Tamura et al. (2024) (70)	*n* = 494	Retrospective	Yes	TEG	\	\	CKH-R > 10 min	FFP: 4 units	CRT MA < 48 mm AND CFF MA > 12 mm	PLT concentrate: 15–20 units	\	\
Naguib et al. (2023) (71)	*n* = 68	Retrospective	Yes	ROTEM	FIBTEM Al0 < 9 mm	Cryoprecipitate: FIBTEM A10:8–9 mm → 1 unit; FIBTEM A10: 7–8 mm → 2 units; FIBTEM: A10 < 7 mm → 3 units	EXTEM CT > 111 s	FFP: 5–l0 ml/kg OR repeat K-centra dose at 20 unit/kg	FIBTEM Al0 > 9 mm	PLT: EXTEM A10: 30–40 mm → 20 ml/kg; EXTEM A10: 20–30 mm → 30 ml/kg; EXTEM A10 < 20 mm → 40 ml/kg	\	\
Li et al. (2023) (37)	*n* = 49	Retrospective	Yes	ROTEM	EXTEM A10 40 mm < 40 + FIBTEM A10 < 10 mm	FC: 5 mg/kg OR croprecipiate 10 units	EXTEM CT > 80 s OR HEPTEM CT > 240 s	PCC: 10–20 U/kg OR FFP: 10–20 ml/kg	EXTEM A0 < 40 mm + FIBTEM A10 > 10 mm	PLTs one dose	\	\
Lanigan et al. (2022) (36)	*n* = 68	Retrospective	Yes	TEG	K > 3 min	Cryoprecipitate: 1 unit (5 U bag)	R: 10–12 min	FFP: 2 units	MA < 47 mm	PLT: 1 unit	\	\
					α angle < 53 °	Cryoprecipitate: 1 unit (5 U bag)	R: 13–15 min	FFP: 3 units	MA < 42 mm	PLT: 2 units		
					\	\	R > 15 min	FFP: 4 units	\	\		
Keyl et al. (2022) (72)	*n* = 248	Retrospective	NA	ROTEM	FIBTEM A10 < 10 mm, FC < 200 md/dl	Fibrinogen	\	\	EXTEM A10 < 40 mm AND PLT count < 100 × 10^3^/μl	PLTs	\	\
Wong et al. (2020) (73)	*n* = 243	RCT	NA	TEG	Functional fibrinogen < 2 g/L	FF < 2 g/L: cryoprecipitate 1 U/30 kg; FF < 1 g/L: cryoprecipitate 1 U/15 kg	R > 11 min	R 11–14 min: FFP 7 ml/kg (1 U/35 kg); R > 14 min: FFP 15 ml/kg (2 U/35 kg)	MA < 50 mm	MA < 40 mm OR suspected PLT dysfunction: PLT 2 units; MA: 40–50 mm PLT 1 unit	\	\
Monaco et al. (2020) (39)	*n* = 40	Retrospective	Yes	ROTEM	FIBTEM A10 < 10 mm	Fibrinogen: 2 g	EXTEM CT > 80 s	PCC: 25 U/kg OR plasma: 15–20 ml/kg	FIBTEM A10 > 10 mm	1 PLT pool	\	\
							INTEM CT > 240 s AND HEPTEM CT = INTEM CT	PCC: 25 U/kg OR plasma: 15–20 ml/kg				
Lehmann et al. (2019) (38)	*n* = 100	RCT	No	ROTEM	EXTEM CFT OR MCF abnormal AND FIBTEM MCF < 6 mm	Fibrinogen: 2 g	INTEM CT ≥ 270 s AND CT HEPTEM > 300 s	FFP: 10 ml/kg body weigh	EXTEM CFT OR MCF abnormal AND FIBTEM MCF >6 mm	PLT concentrate: 1 unit	EXTEM Ll30 < 90% AND APTEM Ll30 normal	TXA: 0.5 g
Haensig et al. (2019) (74)	*n* = 104	RCT	No	ROTEM	FIBTEM MCF < 8 mm	Fibrinogen: 2 g	HEPTEM CT > 260 s	FFP units: FFP (15 ml/kg): < 58 kg BW → 3 FFP; 58–75 kg BW → 4 FFP; 75–92 kg BW → 5 FFP; > 92 kg BW → 6 FFP	HEPTEM MCF: 35–45 mm AND FIBTEM MCF > 8 mm	1 PLT Concentrate	APTEM MCF/HEPTEM MCF > 1.5	TAX: 2 g
									HEPTEM MCF < 35 mm			
Monaco et al. (2019) (41)	*n* = 547	Retrospective	Yes	ROTEM	EXTEM MCF < 45 mm AND/OR INTEM MCF < 45 mm AND A10 FIBTEM > 8 mm	Fibrinogen: 2 g	EXTEM CT > 80 s	Plasma: 15–20 ml/kg OR HEPTEM CT = CT INTEM	EXTEM MCF < 45 mm AND/OR INTEM MCF < 45 mm AND FIBTEM A10 > 10 mm	PLTs: 1 unit	\	\
St-Onge et al. (2019) (40)	*n* = 385	Retrospective	Yes	ROTEM	FIBTEM MCF ≤ 7 mm	Cryoprecipitates: 8 U OR fibrinogen: 2 g	EXTEM pathologic CT, CFT, MCF AND normal right ventricle	FFP: 15 ml/kg	EXTEM and INTEM MCF ≤ 40 mm AND PLTs ≤ 100,000/μl	Five PLTs concentrates (if clopidogrel consider even if PLT > 100 k /μl)	\	\
							EXTEM pathologic CT, CFT, MCF AND abnormal right ventricle	PCC: 15 U/kg				
Rigal et al. (2019) (75)	*n* = 1,000	RCT	NA	ROTEM	EXTEM A10 and HEPTE A10 < 40 mm OR FIBTEM A10 ≤ 10 mm OR fibrinogen < 2 g/L	Fibrinogen: 20–50 mg/kg	FIBTEM A10 > 10 mm and HEPTEM CT > 240 s AND EXTEM CT < 80 s OR EXTEM CT > 80 s need for volume expansion	FFP: 10–15 ml/k	EXTEM A10 and HEPTEM A10 < 40 mm OR FIBTEM A10 > 10 mm OR PLTs < 100 g/L OR antiplatelet drug	PLT 1 pool	EXTEM A10 < 35 mm OR FIBTEM CT > 600 s OR EXTEM ML > 15% OR FIB TEM ML > 10%	TXA: 25 mg/kg
							FIBTEM A10 > 10 mm and HEPTEM CT > 240 s OR PT < 50% AND EXTEM CT > 80 s Needn't for volume expansion	PCC: 12.5 lU/kg				
				TEG	CFF MA < 17 mm and CRT MA > 47 mm OR fibrinogen < 2 g/L	Fibrinogen: 20–50 mg/kg	CKR > 10 min CK-HEP-R >10 min OR PT < 50% need for volume expansion	FFP: 10–15 ml/k	CRT MA < 48 mm and CFF MA > 16 mm OR PLTs < 100 g/L OR antiplatelet drug	PLT 1 pool	CRT LY30 > 3%	TXA: 1–2 g
							CKR > 10 min and CK- HEPTEM-R >10 min OR PT < 50% needn't for volume expansion	PCC: 12.5 lU/kg				
Kuiper et al. (2019) (76)	*n* = 355	Prospective observational	Yes	ROTEM	EXTEM A10 ≤ 40 mm AND HEPTEM A10 ≤ 8 mm	Fibrinogen 2–4 g OR FFP: 10–15 ml/kg	EXTEM CT ≥ 90 s OR FIBTEM CT ≥ 280 s	FFP:10–15 ml/kg OR PCC: 20–30 IE/kg	EXTEM A10 ≤ 40 mm AND FIBTEM A10 ≥ 10 mm	Thrombocyte	\	\
Ichikawa et al. (2018) (77)	*n* = 137	Retrospective	Yes	ROTEM	\	\	EXTEM MCF 50 mm AND FIBTEM MCF > 9 mm	FFP: 3–4 ml/kg needed to increase FIBTEM MCF by 1 mm for a target 15 mm	EXTEM MCF: 50 mm AND FIBTEM MCF > 9 mm	PLTs	\	\
							EXTEM MCF < 50 mm AND/OR FIBTEM MCF < 9 mm	FFP: 3–4 ml/kg needed to increase FIBTEM MCF by 1 mm for a target 10 mm				
Bhardwaj et al. (2018) (78)	*n* = 105	Prospective observational	Yes	ROTEM	EXTEM A10 ≤ 42 mm and FIBTEM A10 ≤ 7.5 mm OR TEG α angle < 48 ° OR SON CR < 13.0	Cryoprecipitate: 1 U/10 kg	EXTEM CT > 95 s INTEM CT > 300 s OR TEG R > 10 min	FFP: 1,015 ml/kg	EXTEM A10 ≤ 42 mm and FIBTEM A10 ≥ 7.5 mm OR TEG MA < 50 mm OR SON PF < 0.9	PLT concentrate: 0.1–0.2 U/Kg	LY 30 < 90%	Antifibrinolytic EACA
Smith et al. (2017) (79)	*n* = 93	Retrospective	Yes	ROTEM	EXTEM A10 ≤ 40 mm and FIBTEM A10 ≤ 9 mm	Cryoprecipitate	EXTEM CT > 90 s OR HEPTEM CT > 280 s	PCC/FFP	EXTEM A10 ≤ 40 mm and FIBTEM A10 ≥ 10 mm	PLTs/DDAVP	Lysis index LI 30 > 15 %	TXA
Nakayama et al. (2015) (34)	*n* = 100	RCT	Yes	ROTEM	\	\	EXTEM A10 > 30 mm AND FIBTEM A10 ≤ 5 mm	FFP: 20 ml/kg	EXTEM A10 ≤ 30 mm AND FIBTEM A10 > 5 mm	PLT: 10 ml/kg	\	\
Karkouti et al. (2015) (35)	*n* = 2,481	Retrospective	Yes	ROTEM	FIBTEM A10 ≤ 7 mm	Cryoprecipitate: 10 U	EXTEM CT ≥ 100 s	Plasma: 10–15 ml/kg BW	EXTEM A10 < 35 mm AND FIBTEM A10 > 7 mm	PLTs 1 pool	\	\
Faraoni et al. (2015) (80)	NA	Retrospective	NA	ROTEM	FIBTEM A10 ≤ 3 mm	FC: 25 mg/kg	EXTEM CT ≥ 111 s	FFP: 15 ml/kg	EXTEM A10 ≤ 38 mm AND FIBTEM A10 > 3 mm	PLTs 1 Unit/5 k	\	\
Westbrook et al. (2009) (81)	*n* = 60	RCT	YES	TEG	α angle < 45 °	Cryoprecipitate: 5 units	R > 16 min	FFP: 4 units	MA (H) ≤ 41 mm	PLT 5 unites	LY30 > 15%	TXA
Shore-Lesserson et al. (1999) (82)	*n* = 105	RCT	YES	TEG	\	\	hTEG R > 20 mm	FFP	PLT Count < 100 K AND MA < 45 mm	Platelets	TEG LY30 > 7.5%	EACA

FC, fibrinogen concentrate; FFP, fresh frozen plasma; PCC, prothrombin complex concentrate; PLT, platelet; TXA, tranexamic acid; FIBTEM, fibrinogen thromboelastometry; EXTEM, external thromboelastometry; INTEM, intrinsic thromboelastometry; HPTEM, heparinase thromboelastometry; CT, time from test start to an amplitude of 2 mm; CFF, citrated functional fibrinogen; CRT, citrated rapid TEG; LI30, percentage of clot lysis at 30 min; LY30, percentage of clot lysis at 30 min; A10, the clot amplitude at 10 min after the beginning of clot formation; MA, maximum clot strength; hTEG, heparinase-activated; CKR, citrated kaolin test reaction time; K, time between 2 and 20 mm clot amplitude; α angle, slope between R and K; CFT, clot formation time; MCF, maximum clot firmness; ML, maximum lysis; FF, functional fibrinogen; CK-HEP-R, citrated kaolin heparinase test reaction time; CKH-R, heparinase kaolin reaction time; SON, sonoclot, it is one of viscoelastic hemostatic assay; CR, clot rate; PF, platelet function; PT, prothrombin time; EACA, epsilon amino caproic acid.

Based on the aforementioned trigger thresholds, a randomized controlled trial by Nakayama et al. (34) demonstrated that ROTEM-guided management of blood products post-bypass surgery, compared to an algorithm based on ACT and platelet count (conventional laboratory measurements), reduced postoperative bleeding at 12 h (9 vs. 16 ml/kg, *p* = 0.001), the need for packed RBCs transfusions (11 vs. 23 ml/kg, *p* = 0.005), and intensive care unit stay duration (60 vs. 71 h, *p* = 0.014). A retrospective cohort study of 2,481 patients by Karkouti et al. (35) showed that the use of ROTEM was significantly associated with reduced transfusion rates of RBCs (OR = 0.50; 95% CI: 0.32–0.77; *p* = 0.002), platelets (OR = 0.22; 95% CI: 0.13–0.37; *p* = 0.002), and plasma (OR = 0.20; 95% CI: 0.12–0.34; *p* = 0.002). Similarly, a retrospective study by Lanigan et al. (36) that analyzed 68 patients who underwent left ventricular assist device implantation or heart transplantation showed reductions in absolute units of FFP, platelets, and cryoprecipitate by 40.2, 47.5, and 63.3%, respectively, along with significant decreases in transfusion rates and extubation time. However, Li et al. (37) and Lehmann et al. (38) showed that VHA-guided blood management did not significantly reduce the bleeding and transfusion of blood products like RBCs and FFP in cardiac surgery.

For major arterial surgeries, Monaco et al. (39) conducted a retrospective study on 40 patients who selectively underwent aortic arch replacement with the frozen elephant trunk technique; the results showed that ROTEM-guided treatment reduced the infusion of 1,345 ml of plasma and 0.91 units of platelets vs. conventional coagulation tests. A retrospective analysis by St-Onge et al. (40) on 385 patients undergoing aortic surgery using ROTEM showed a reduction in RBCs transfusion units [1.0 (0.0–4.0) units vs. 0.0 (0.0–2.0) units, *p* = 0.03] and a decrease in FFP transfusion [0.0 (0.0–4.0) units vs. 0.0 (0.0–2.0) units, *p* = 0.04], and was significantly associated with reduced massive transfusions (*p* = 0.04) and shorter Intensive Care Unit stays (*p* < 0.01). Additionally, a retrospective analysis by Monaco et al. (41) on 547 patients undergoing open repair of thoracoabdominal aortic aneurysms showed that patients managed with ROTEM rather than conventional coagulation tests had reduced transfusion of RBCs and FFP, with significantly fewer patients receiving FFP (*p* < 0.001); furthermore, the incidence of pulmonary complications (44 vs. 83%; *p* = 0.01) and medical costs were also reduced. Therefore, using VHA in major vascular surgeries can reduce the transfusion of blood products, decrease postoperative complications, and improve patient outcomes.

## VHA in postpartum hemorrhage

Postpartum hemorrhage (PPH) is one of the most dangerous postpartum complications, which can be caused by uterine atony, retained placenta, and birth canal lacerations (42). During pregnancy, the increase in coagulation factors disrupts the balance of bleeding, clotting, and fibrinolysis in the blood system (43), leading to varying degrees of bleeding and postpartum thromboembolic events (44), therefore, timely and accurate identification of coagulation status and transfusion intervention in patients with PPH is very important. Studies have shown that VHA is advantageous in diagnosing coagulopathy, monitoring fibrinolysis in women with obstetric hemorrhage, predicting PPH, and reducing transfusions (45), but most studies have low certainty of evidence and are biased to be widely used in clinical practice.

The algorithm for VHA-guided blood management in patients with PPH is shown in Table 4. Researchers initiate fibrinogen and other transfusions when ROTEM FIBTEM A5 is < 7–12 mm, alone or in combination with EXTEM A5 < 35 or 47 mm; in TEG, primarily when (CFF) MA is < 14–19 mm, A10 is ≤ 17 mm, or the α-angle is < 45 (45, 46). When EXTEM CT ≥ 75–100 s or TEG R ≥ 9–12 mins, FFP or PCC transfusion is administered. In addition, platelet transfusion is performed when EXTEM A5 < 35 mm or A10 < 47 mm and FIBTEM A5 > 11–13 mm, TEG MA < 48–57 mm (45, 46). For TXA, the trigger threshold is rarely mentioned because patients with PPH are generally given a standard intravenous infusion of 1 g of TXA.

**Table 4:** Trigger thresholds and transfusion in postpartum hemorrhage using VHA algorithms.

**Author (year)**	**Sample size**	**Research type**	**Reduction of blood product**	**VHA**	**FC/fibrinogen/cryoprecipitate**		**FFP/PLASMA/PCC**		**PLT**		**TXA**	
					**Trigger value**	**Dosage**	**Trigger value**	**Dosage**	**Trigger value**	**Dosage**	**Trigger value**	**Dosage**
Jokinen et al. (2023) (8)	*n* = 60	RCT	Yes	ROTEM	EXTEM A5 < 35 mm AND FIBTEM A5 < 12 mm	FC: 3–4 g	EXTEM CT > 80 s	70 kg: 5 Octaplas units; 90 kg: 7 octaplas units OR 70 kg: PCC 60 ml; 90 kg: PCC 70 ml	EXTEM A5 < 35 mm AND FIBTEM A5 > 12 mm	PLTs: 2 bags	EXTEM A5 > 35 mm AND EXTEM ML > 5%	Repeating bolus of TXA 1 g
Yurashevich et al. (2023) (47)	*n* = 165	Retrospective	NA	ROTEM	EXTEM A10 < 45 mm AND FIBTEM A10 < 13 mm	Cryoprecipitate: 1 bag (5 U) OR FC: 2 g	EXTEM CT > 80 s	FFP: 10–15 ml/kg OR PCC (Kcentra): 4 F	EXTEM A10 < 45 mm AND FIBTEM A10 > 13 mm	PLT: 1 bag	\	TXA 1 g IV (bolus)
					EXTEM A10 < 45 mm AND FIBTEM A10 < 10 mm	Cryoprecipitate: 2 bag (10 U) OR FC: 4 g	Refractory bleed	PCC				TXA 1 g infusion 1 mg/kg/h
Mallaiah et al. (2015) (49)	*n* = 93	Retrospective	Yes	ROTEM	FIBTEM A5 < 7 mm AND EXTEM A5 < 47 mm	FC: 3 g	EXTEM CT > 100 s AND Active bleeding	FFP	Low EXTEM (but normal FIBTEM OR if 10 units of blood OR more)	PLTs	\	\
					FIBTEM A5: 7–12 mm AND EXTEM A5 < 47 mm active/high risk of bleeding	FC: 3 g	EXTEM CT > 100 s AND Active bleeding					

FC, fibrinogen concentrate; FFP, fresh frozen plasma; PCC, prothrombin complex concentrate; PLT, platelet; TXA, tranexamic acid; FIBTEM, fibrinogen thromboelastometry; EXTEM, external thromboelastometry; A10, the clot amplitude at 10 min after the beginning of clot formation; A5, the clot amplitude at 5 min after the beginning of clot formation; CT, time from test start to an amplitude of 2 mm.

In the randomized controlled trial by Jokinen et al. (8) where 54 women with PPH were divided into a ROTEM-guided group and a conventional treatment group, the results showed that the ROTEM-guided transfusion algorithm reduced plasma usage (0–0 vs. 0–2, *p* = 0.030) and total blood loss [2,500 ml (2,100–3,000) vs. 3,000 ml (2,200–3,100), *p* = 0.033]. However, there are few studies on using VHA to manage RBCs or PLT transfusion in PPH, with most research focusing on using VHA to guide FC or FFP.

A retrospective study by Yurashevich et al. (47) demonstrated that reduced fibrinogen levels are independently associated with severe hemorrhage. Similarly, Agarwal et al. (48) showed that fibrinogen levels in obstetrics can predict massive hemorrhage, with fibrinogen being the first to decrease during massive hemorrhage. Therefore, an early identification of reduced fibrinogen levels is crucial. Mallaiah et al. (49) demonstrated that a reduction in ROTEM FIBTEM A5 and EXTEM A5 indicates a decrease in fibrinogen and can guide the use of FC. McNamara et al. (50) analyzed 4 years of observational data, showing that FIBTEM A5 < 7, or 7–12 mm, indicates a risk of ongoing or severe bleeding, and using the VHA algorithm can reduce the number of blood product units transfused (*p* < 0.0001) and the total volume (*p* = 0.0007), as well as decrease transfusion-associated circulatory overload (*p* = 0.002). Additionally, an observational study by Collins et al. (51) on 605 postpartum women demonstrated that using FFP to control FIBTEM A5 < 15 mm is feasible in actual clinical practice. Therefore, in obstetrics, the VHA algorithm is widely used to guide the use of FC, aiming to prevent and reduce bleeding.

## VHA in liver surgery

Liver transplantation has become the primary treatment for end-stage liver disease (52). In advanced cirrhosis and post-liver transplantation, reduced clotting factors establish a new but unstable hemostatic equilibrium, creating dual risks: increased thrombosis resulting from this delicate balance and bleeding risk caused by portal hypertension. Therefore, managing each component of the blood system is necessary to prevent major bleeding events. While the use of VHA is common in liver surgery, there is still a controversy regarding the trigger thresholds for using VHA in cases of major bleeding in liver surgery, as well as its impact on reducing transfusions and improving patient prognosis and mortality. This is due to factors such as the inherent complexity of coagulation in liver surgery patients, differences in patient types, and the generally low quality of evidence in related studies.

Table 5 outlines the algorithm for VHA-guided blood management in patients with severe bleeding and those at risk of severe bleeding due to liver surgery. The management is mainly guided by ROTEM FIBTEM A10 ≤ 8 mm alone or combined with EXTEM A5 or A10; TEG MA < 30 mm and fibrinogen functional MA (FF MA) ≤ 7 mm, as well as TEG α angle < 45 °, to guide the transfusion of fibrinogen, FC and cryoprecipitate, to achieve fibrinogen supplementation purposes (53, 54). FFP or PCC transfusion is indicated when EXTEM CT > 100 s or > 80–90 s, or combined with INTEM CT and HEPTEM CT, or TEG R > 14–40 min. In ROTEM, platelet transfusion is triggered by EXTEM A10, A5, and MFC combined with FIBTEM A10 > 8 or 9 mm. TEG indicates platelet transfusion when MA < 30 or 40–48 mm. Additionally, TXA infusion primarily focuses on TEG Ly30 > 7.5% or ROTEM ML, CTF, or MCF.

**Table 5:** Trigger thresholds and transfusion in liver surgery using VHA algorithms.

**Author (year)**	**Sample size**	**Research type**	**Reduction of blood product**	**VHA**	**FC/fibrinogen/ cryoprecipitate**		**FFP/PLASMA/PCC**		**PLT**		**TXA**	
					**Trigger value**	**Dosage**	**Trigger value**	**Dosage**	**Trigger value**	**Dosage**	**Trigger value**	**Dosage**
Janko et al. (2023) (83)	NA	RCT	NA	ROTEM	EXTEM A5 < 35 mm AND FIBTEM A5 < 9 mm	One dose cryoprecipitate (5 × 60 ml or 10 × 35 ml)	EXTEM CT > 80 s AND cryoprecipitate also indicated	1 unit FFP	EXTEM A5 < 35 mm AND FIBTEM A5 ≥ 9 mm	1–2 bags PLTs	\	\
							EXTEM CT > 80 s AND no cryoprecipitate indicated	FFP: 10–15 ml/kg				
Premkumar et al. (2021) (84)	*n* = 142	Prospective observational	Yes	TEG	\	\	R > 14 mm AND < 21 mm	FFPs: 2 units	MA < 48 mm	1 SDP (single-donor PLT) OR 4 RDPCs (random donor PLT concentrate)	Lys30 > 7.5%	TXA
							R > 21 mm AND < 28 mm	FFPs: 4 units	MA < 40 mm	2 SDPs OR 68 RDPCs		
							R > 28 mm	FFPs: 6–8 units	\	\		
Bonnet et al. (2019) (57)	*n* = 81	RCT	No	ROTEM	FIBTEM A10 ≤ 8 mm	Fibrinogen: 3 g	EXTEM CT > 110 s	FFP: 2 units	EXTEM MCF < 40 mm OR A10 < 35 mm AND FIBTEM A10 OR MCF > 8 mm	PLT 1 unit	EXTEM hyperfibrinolysis OR maximal lysis > 15%	TXA:1 g bolus then 3 g daily
											APTEM decrease > 15% CT OR CFT OR increase > 15% MCF compared with EXTEM	
Smart et al. (2017) (56)	*n* = 68	Prospective observational	Yes	ROTEM	FIBTEM MCF < 10 mm	cryoprecipitate: 1–2 U	EXTEM CT > 90 s	FFP: 4 units	INTEM AND EXTEM MCF < 50 mm	1–2 units PLTs	INTEM AND EXTEM ML > × 15% AND CI < 1.0	Aminocaproic acid: 2 g
De Pietri et al. (2016) (59)	*n* = 60	RCT	Yes	TEG	\	\	R > 40 min	FFP	MA < 30 mm	PLT	\	\
De Pietri et al. (2016) (58)	*n* = 386	Retrospective	Yes	TEG	MA < 30 mm AND FF MA ≤ 7 mm	FC: 25–50 mg/kg	R > 40 min AND FF MA > 7 mm	FFP: 15–20 ml/kg	MA < 30 mm AND FF MA > 7 mm	PLT apheresis 1 U	Fibrinolysis	TXA: 10–15 mg/kg
Meserve et al. (2015) (85)	NA	RCT	NA	ROTEM	FBTEM MCF < 10 mm	1 U cryoprecipitate (pooled from 5 U Plasma)	EXTEM CT > 85 s OR INTEM CT AND HEPTEM CT > 210 s	2 U FP (6–7 ml/Kg)	PLTs < 50,000/cumm AND any prolongation of CT OR Prolonged CT AND FIBTEM MCF < 10 mm	High risk OF bleeding: 2 U cryoprecipitate +/– PLTs +/−1–2 U frozen plasma if FV AND/OR FVll < 30%	\	\

FC, fibrinogen concentrate; FFP, fresh frozen plasma; PCC, prothrombin complex concentrate; PLT, platelet; TXA, tranexamic acid; FIBTEM, fibrinogen thromboelastometry; EXTEM, external thromboelastometry; INTEM, intrinsic thromboelastometry; HPTEM, heparinase thromboelastometry; APTEM, aprotinin thromboelastometry; A10, the clot amplitude at 10 min after the beginning of clot formation; A5, the clot amplitude at 5 min after the beginning of clot formation; CT, time from test start to an amplitude of 2 mm; ML, maximum lysis; MCF, maximum clot firmness; MA, maximum clot strength; R, time from test start to an amplitude of 2 mm; FP, frozen plasma; FF, functional fibrinogen; CFT, clot formation time; CI, clot index; Lys30, lysis at 30 min; SDPs, single-donor platelets; RDPCs, random donor platelet concentrates.

The use of VHA for blood management and treatment in liver transplantation is widespread (53, 55). A randomized controlled trial by Smart et al. (56) involving 64 orthotopic liver transplantation (OLT) patients showed that ROTEM-guided therapy, compared to conventional coagulation tests, reduced intraoperative blood loss (2.0 vs. 3.0 L, *p* = 0.04) and FFP transfusions (4 vs. 6.5 units, *p* = 0.015), but increased cryoprecipitate use (73% vs. 56%, *p* = 0.033). Bonnet et al.'s (57) randomized study of 81 OLT patients found that replacing standard coagulation test algorithm with ROTEM reduced transfusion requirements and FFP use, though fibrinogen transfusion increased (72.5 vs. 29.3%, *p* < 0.001). Similarly, De Pietri et al.'s (58) retrospective study of 386 liver transplant patients showed that FF-TEG testing, rather than the previously used TEG-based algorithm, significantly reduced blood, FFP, and platelet transfusions while increasing fibrinogen administration. This suggests that VHA can reduce the use of blood products other than fibrinogen.

Patients with advanced liver cirrhosis face the risk of coagulation system disorders, which can lead to life-threatening bleeding during interventional treatments. Therefore, VHA algorithms are particularly necessary to guide pre-intervention blood management in these patients. The study by De Pietri et al. (59) demonstrated that using TEG to guide transfusions before interventional treatment for liver cirrhosis can reduce the use of FFP and PLT. Maria et al. (60) support this viewpoint, and their randomized controlled study using the ROTEM algorithm on 60 patients undergoing interventional treatment for liver cirrhosis demonstrated not only a reduction in the use of FFP and PLT but also a decrease in overall blood product utilization (46.7 vs. 100%, *p* < 0.001), highlighting its cost-effectiveness. Therefore, before interventional surgery for liver cirrhosis, clotting function management guided by VHA algorithms should be conducted to optimize treatment protocols.

## VHA in pediatric surgery

Due to underdeveloped immune and coagulation systems, inadequate temperature regulation, and lower tolerance to post-major surgery stress, pediatric patients are more prone to complications such as bleeding after a major surgery. Therefore, many researchers apply VHA in pediatric patients to enhance the management of post-major surgery bleeding. Nevertheless, the clinical use of VHA for guiding transfusion trigger thresholds in pediatric patients undergoing major surgery is still uncommon, and the level of evidence from relevant studies is insufficient to be used for generalization, with each study's trigger thresholds having their own characteristics, making it difficult to unify them.

A randomized controlled study by Haas et al. (61) using fibrinogen concentrate (FC) under the ROTEM algorithm to reduce RBCs transfusion demonstrated that triggering FC use with ROTEM FIBTEM < 13 mm can reduce bleeding and transfusion requirements in pediatric patients undergoing craniosynostosis surgery. A randomized controlled study by Zhang et al. (62) on 83 pediatric patients undergoing resective epilepsy surgery showed that the prophylactic use of TXA combined with the TEG algorithm significantly reduced the transfusion rates by 34.7% (*p* = 0.001), primarily through a decrease in plasma transfusions, though there was an increase in FC transfusion rates. Similarly, Raffaeli et al. (63) also believe that TEG can reduce the use of FFP, and experimental verification showed that TEG use in neonatal surgery decreased the use of FFP (31 vs. 60%, *p* < 0.001). However, the algorithm for using VHA in pediatric patients is still not fully developed, and further research and precision are needed regarding the trigger thresholds for VHA. Nevertheless, it is undeniable that its use in pediatric patients has optimized intraoperative blood management.

## Conclusions

In various fields such as trauma, cardiac, postpartum hemorrhage, hepatic, and pediatrics, there is a lack of tools for timely and accurate assessment and transfusion intervention in patients with massive transfusion risk, thus making VHA a clinically worthwhile transfusion algorithm. Most studies indicate that VHA transfusion trigger thresholds exhibit significant heterogeneity due to different populations and research methodologies. Therefore, establishing different VHA trigger thresholds for different populations holds epoch-making significance for the development of viscoelastic hemostatic technology, the resolution of clinical problems, and clinical research.

While the trigger thresholds of VHA algorithms vary across different types of surgeries, there are still commonalities TEG and ROTEM parameters within the same population. The transfusion of fibrinogen, FC, and cryoprecipitate is primarily determined by ROTEM FIBTEM A5, A10, or MCF, TEG α-angle, or MA. FFP and PCC transfusions are guided mainly by ROTEM EXTEM CT and TEG R. Additionally, platelet transfusions are mostly guided by ROTEM EXTEM combined with FIBTEM A5, A10, or MCF, or TEG MA. The administration of TXA is primarily determined by ROTEM ML, LI30, or TEG Ly30.

VHA is now used in various surgeries involving severe hemorrhage and potential coagulopathy; however, clinical adherence to VHA-guided blood product transfusion remains low. Therefore, while there are challenges in the current practical application of using VHA to assess patients' coagulopathy risk and guide clinical transfusion decisions, the future prospects are promising. Based on factors such as poor historical compliance with VHA guidance, many studies suffer from biases, omissions, insufficient sample sizes, and other issues, resulting in insufficient evidence strength for many VHA studies. Despite this, many guidelines still recommend using VHA to guide blood management and fluid resuscitation (20–23). Furthermore, an increasing number of researchers are exploring using VHA algorithms to guide transfusion in various types of major bleeding or coagulation disorder surgeries. These studies further refine and validate the VHA algorithms, demonstrating that VHA-guided management facilitates early diagnosis and prevention of coagulopathy, reduces bleeding and the use of blood products (RBCs, FFP, FC, and PLT), and can lower the incidence of transfusion-related complications to improve prognosis. To address these issues, we should conduct education and training on VHA usage, standardize clinical workflows, and reduce VHA instrument costs to achieve widespread adoption and increased adherence.

At the same time, viscoelastic hemostasis technology continues to evolve, with the emergence of new tools for dynamic measurement of coagulation status, including Sonoclot (64), ClotPro (65, 66), SEER (67) and Quantra QPlus systems (68), in addition to the commonly used TEG and ROTEM. They significantly reduce measurement time, broaden the range of applications, and increase the accuracy of measurements (69).

VHA-guided blood management facilitates the management and treatment of major bleeding, improves patient's quality of life, and conserves medical resources. However, research on VHA-guided transfusion algorithms is still far from sufficient. Alongside standardizing VHA utilization among clinicians, extensive prospective randomized multicenter studies and multidisciplinary collaboration are essential to explore whether universal thresholds can be established or whether VHA algorithm trigger thresholds can be optimized for different surgical types and individualized intraoperative and postoperative major bleeding scenarios, while demonstrating the clinical benefits of VHA algorithms. Furthermore, comparative studies of various VHA devices are needed to identify economical, rapid, and accurate VHA instruments with their corresponding thresholds for perioperative application.
